# Ventilator-induced lung-injury in mouse models: Is there a trap?

**DOI:** 10.1186/s42826-021-00108-x

**Published:** 2021-10-29

**Authors:** Jon Petur Joelsson, Saevar Ingthorsson, Jennifer Kricker, Thorarinn Gudjonsson, Sigurbergur Karason

**Affiliations:** 1 Stem Cell Research Unit, BioMedical Center, School of Health Sciences, University of Iceland, Reykjavik, Iceland; 2 Department of Laboratory Hematology, Landspitali-University Hospital, Reykjavik, Iceland; 3 Faculty of Nursing, University of Iceland, Reykjavik, Iceland; 4 EpiEndo Pharmaceuticals, Seltjarnarnes, Iceland; 5 Intensive Care Unit, Landspitali-University Hospital, Reykjavik, Iceland

**Keywords:** Ventilator-induced lung injury, Mouse studies, Animal models, Acute lung injury

## Abstract

Ventilator-induced lung injury (VILI) is a serious acute injury to the lung tissue that can develop during mechanical ventilation of patients. Due to the mechanical strain of ventilation, damage can occur in the bronchiolar and alveolar epithelium resulting in a cascade of events that may be fatal to the patients. Patients requiring mechanical ventilation are often critically ill, which limits the possibility of obtaining patient samples, making VILI research challenging. In vitro models are very important for VILI research, but the complexity of the cellular interactions in multi-organ animals, necessitates in vivo studies where the mouse model is a common choice. However, the settings and duration of ventilation used to create VILI in mice vary greatly, causing uncertainty in interpretation and comparison of results. This review examines approaches to induce VILI in mouse models for the last 10 years, to our best knowledge, summarizing methods and key parameters presented across the studies. The results imply that a more standardized approach is warranted.

**Supplementary Information:**

The online version contains supplementary material available at 10.1186/s42826-021-00108-x.

## Background

Mechanical ventilation (MV) of patients may be required for various reasons but the main applications in critically ill patients are alleviating work of breathing and ensuring sufficient gas exchange during the resolution of the underlying disease. It has been established, however, that ventilation treatment may harm the lung and increase mortality [1].

During normal inspiration, air flows into the lungs due to negative pressure generated in the thorax by expanding the lungs via contraction of the diaphragm and intercostal muscles. During MV, the opposite occurs, air is thrust into the lungs with the use of positive pressure from the ventilator, which can lead to damage of the lung epithelium due to mechanical strain, named ventilator-induced lung injury (VILI) [2].

**Fig. 1:**
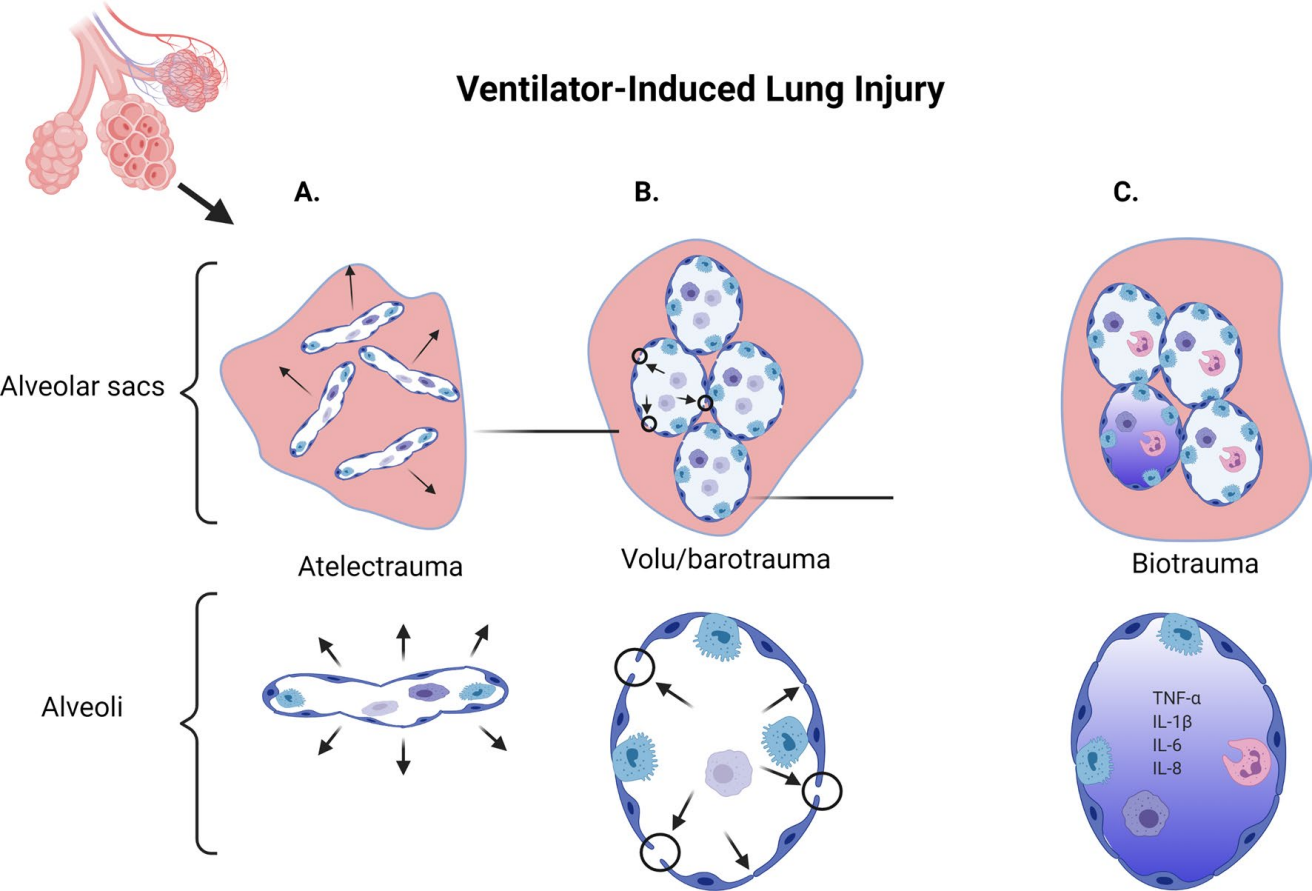
Ventilator-induced lung injury. **A** Mechanical ventilation with insufficient PEEP can lead to collapse of the alveoli, leading to atelectrauma. Inspiration and expiration from the mechanical ventilator will lead to the alveoli being repeatedly opened and collapsed, which will lead to shear stress and injury. **B** Inappropriately high pressure/tidal volume settings from the mechanical ventilator can lead to overdistension of the distal bronchi and alveoli, leading to volu/barotrauma injury and barrier disruption. **C** The damage caused by atelectrauma and volu/barotrauma cumulatively can lead to activation of immune and epithelial cells, causing an excessive inflammatory response which can lead to an influx of inflammatory mediators (TNF-α, IL-1β, IL-6 and IL-8), resulting in what is called biotrauma. Figure created with BioRender.com

Consequently, MV can exacerbate respiratory failure in already diseased lung tissue, or even be the primary cause of it through VILI in healthy lungs if they are recklessly ventilated. If adequate positive end-expiratory pressure (PEEP) is not used, the alveoli will collapse during expiration and then be reopened forcefully and repeatedly with each inspiration rendering them susceptible to shear stress and injury in what is called atelectrauma (Fig. 1A). During inspiration, if pressure and/or volume is inappropriately high, open alveoli may be subjected to over-distension and damaged in what is termed volu/barotrauma (Fig. 1B). Additionally, in severe respiratory failure, a high fraction of oxygen in inspiratory air is usually used that can increase the oxidative stress of the tissue and cause cell injury. Cumulatively, these damage-inducing factors can lead to an initial activation of local immune response in the lung tissue, causing a cascade effect resulting in an excessive immune response and overflow of inflammatory parameters into the bloodstream affecting other organs, in what is termed biotrauma (Fig. 1C) [3].

Clinical research on VILI in patients is challenging due to the high-risk nature of invasive diagnostic procedures in patients with serious respiratory failure. Access to patient lung samples is therefore limited, effectively restricting research to either healthy tissue or end-point samples; emphasizing the need for alternative methodologies such as in vitro research, or in vivo animal models.

In vitro models of VILI are few and physiologically limited. The lung is a complex organ and contains several epithelial, interstitial, and immune cell types arranged in a wide variety of tissues within the organ, which is extremely difficult to recapitulate in vitro [4]. Cell models usually do not adequately represent the mechanical forces that lung tissues and cells in vivo are subjected to. Our laboratory has, however, recently established a novel in vitro system to mimic VILI in lung epithelial cells cultured in air–liquid interface conditions, using the cyclical pressure air–liquid interface device (CPAD) [5]. Employing the CPAD, we were able to mimic important events in VILI by inducing barrier damage in bronchial and alveolar epithelial cell layers. We showed that azithromycin treatment protected the epithelial layer against mechanical strain by maintaining the actin structures of the cytoskeleton, changing lipid metabolism, and attenuating inflammatory responses [6]. In vitro systems are useful to gain an initial indication of relevant mechanisms but they lack the complex epithelium, stroma and the microvasculature interactions. Therefore, translation of results from in vitro studies to clinical usefulness requires testing in in vivo models.

Mice have been extensively used to study VILI. However, there is no standardized model for establishing VILI in mice in the literature and important parameters of ventilator configurations and settings in published papers vary considerably. These key parameters include choice of tidal volume, time of ventilation, breaths per minute, use of protective ventilation strategies such as PEEP, choice of anesthetic drugs, type of animal used, and whether it is appropriate to base the ventilation on pressure or tidal volume generated by the mechanical ventilator. Such variation creates difficulties in reproducing an equivalent, robust model, as well as interpretation and comparison of results.

In 2010, the American Thoracic Society published a workshop report with a consensus of the main features characterizing acute lung injury (ALI) in animal models and identified the most relevant methods to assess these features [7]. These methods were categorized based on relevance regarding tissue injury, capillary barrier function, inflammatory response and physiological dysfunction. The report was intended as a guide for researchers to choose appropriate measurements best suited for their experimental questions.

The purpose of this review is to summarize information from in vivo VILI experiments in mice conducted over the last decade, highlighting the range of ventilator modes and settings and selection of mice strains, then identifying advantages and disadvantages of various experimental set ups. Such information should assist researchers in choosing ventilator configurations appropriate for their studies, enabling a more standardized approach towards certain research questions allowing easier interpretation and comparison of results.

**Fig. 2:**
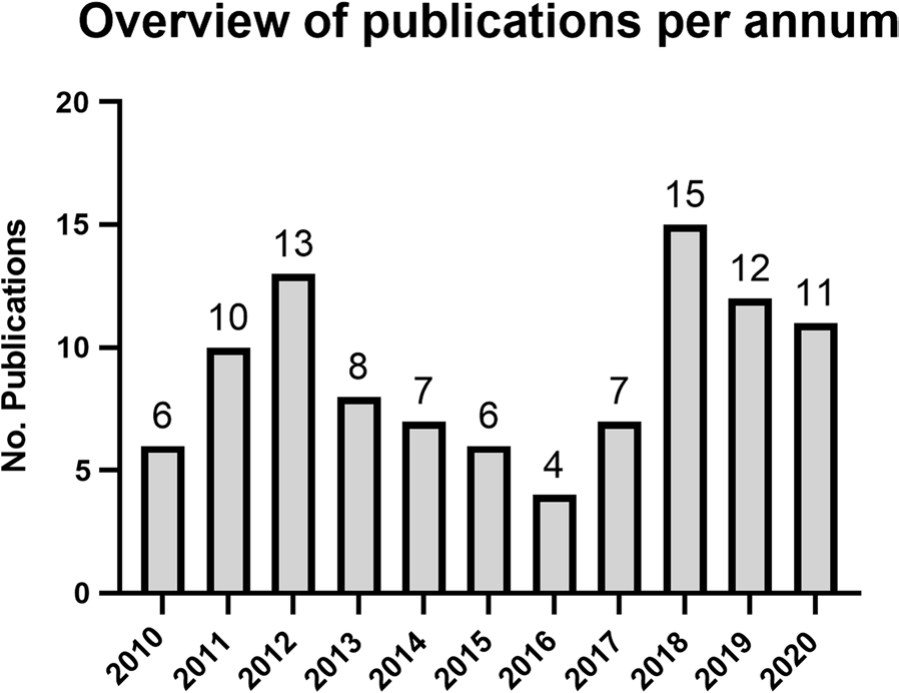
Overview of publications in this review, per annum

A PubMed search was conducted with the search term “ventilator induced lung injury mouse model”, filtering the publication year from 2010 to 2020. From the 372 matching hits, we concentrated on models where mice were mechanically ventilated, leaving a pool of 99 publications [8–106]. Figure 2 shows the distribution of the publications over the 10 years. Each article was reviewed in depth and information on various parameters identified and key information listed in a table (Additional file 1: Table S1).

## Main text

### Mouse strains

Animal models, especially mouse strains, are vital in biomedical research as they bridge basic research from the in vitro to the in vivo clinical setting. There are many mouse strains available for biological research. Inbred mouse strains are genetically homologous, thereby reducing the risk of variation in the results, while outbred stocks such as CD-1 represent heterozygous populations and higher variability in the results. The choice of mouse strain to use for an experimental application depends on the research question being addressed. In the case of lung health, the test animals’ response to stress and damage should not be compromised, but it is critical that the animals are capable of generating a strong immune response, as inflammation is the premise for VILI. In this regard, mouse strains that are immunodeficient, such as CB17SCID mice are preferred for studying cancer biology and cancer treatment strategies, but they are suboptimal for studying normal pathophysiology, for example lung physiology.

**Fig. 3:**
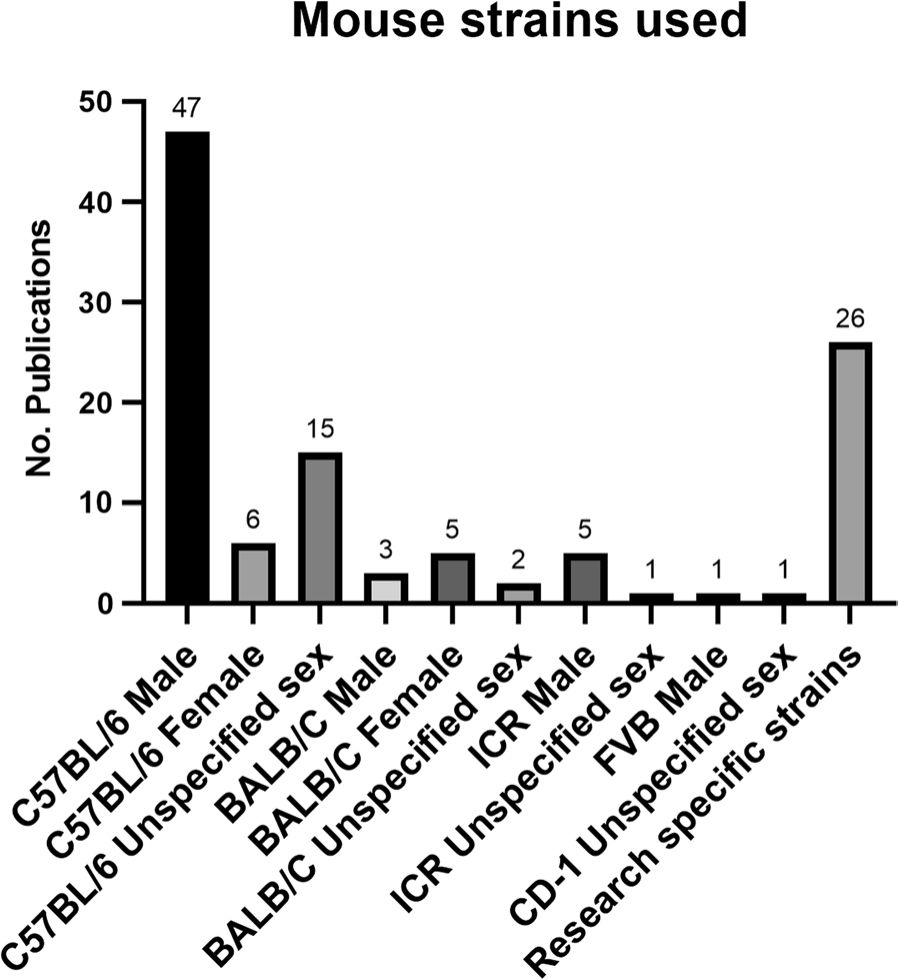
Overview of mouse strains used in the reviewed publications per annum. Often, more than one strain was used

One of the most popular mouse strains for research is the C57BL/6. These black inbred mice are a stable strain and easily bred. They represent the majority of the mice used for VILI research covered in this review (Fig. 3) C57BL/6 mice are known for their immunogenicity as they were initially bred for antitumor and immunological research purposes [107]. Several repositories breed C57BL/6 mice, including Jackson Laboratories (C57BL/6J) and Taconic (C57BL/6Tac). These breeding colonies have been genetically isolated for tens or hundreds of generations and due to genetic drift have become genetically different from one another; a fact that needs to be considered when choosing animals for studies [108]. In the selected papers on VILI, 68 instances of mouse strain selected were for C57BL/6. Of those, 47 were male and 6 female and in 15 studies, the sex was not specified in the methods.

BALB/c mice are also a popular choice for VILI studies. BALB/c mice are an inbred, immunodeficient, albino mouse strain. They are easily bred but tumor-prone [109]. In the publications checked, BALB/c mice were used in 10 instances and of those, 3 were male, 5 were female and 2 were not specified. Other mouse strains to note were ICR, sensitive A/J and CD-1 strains. Twenty-six strains were specifically attained or bred for the VILI research in question. These include TRPM −/−, NLRP3 −/− [82], Src—deficient [55], Tpl2 −/− [47], RAGE KO [48], TNF receptor 2 KO [68], TLR4 KO [66], TRPV4—deficient [65], gpl1 +/− and Sphk1 −/− [76], Zmpste24 −/− and Lmna LCS/LCS [73], IL6 −/− [16, 45], Muc5ac—deficient [39] and Akt +/− [18]. In 4 instances neither the strain nor sex of the mice were specified. A thorough overview over different mouse strains can be found at Labome [110].

**Fig. 4:**
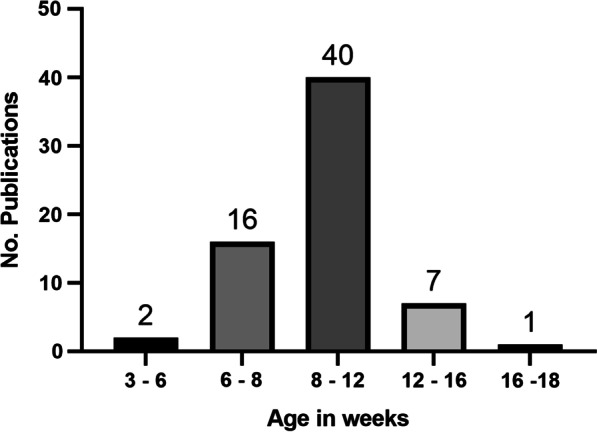
Overview of the age range of mice in the reviewed publications

Besides mouse strain and gender, age was also highly variable. To simplify our summary, we categorized the mice into the following age groups with the number of instances in parentheses: 3–6 weeks (2); 6–8 weeks (16); 8–12 weeks (40); 12–16 weeks (7); 16–18 weeks (1) (Fig. 4). Only one publication used neonatal mice. Although the 8–12 weeks age range, young adult mice, was most widely used, there did not appear to be a consensus of optimal age. Moreover, the age of the mice was not specified in 35 of the publications.

The weight of the mice was largely consistent with their respective ages, but this could not always be ascertained as 49 of the publications on VILI did not state the weight of the mice specifically. The weight range was mostly within 20–25 g with the lowest being 17.1 g and the highest being 30 g.

### Ventilator settings

Positive pressure ventilation has been used to treat patients for more than six decades [111]. However, it is still debated how such treatment can be optimized as there are no clinical methods to individualize the treatment but only empirical guidelines on how to set the ventilator [112]. There has been an emphasis in recent years on using protective lung ventilation (PLV) for all patients undergoing ventilator treatment in order to avoid VILI, even in those with healthy lungs. The purpose of PLV is to keep the mechanical strain as low as possible to prevent VILI using the following principles: (1) using a low tidal volume between 4 and 8 mL/kg according to predicted body weight, (2) keeping peak or plateau pressure below 30 cm H_2_O, (3) setting PEEP according to the severity of the respiratory failure, usually not higher that 10–15 cm H_2_O in adults with severe respiratory failure, (4) keeping the driving pressure (the pressure difference between end-inspiration (plateau pressure) and end-expiration (PEEP)) below 15 cm H_2_O; and (5) adjusting the respiratory rate to keep PaCO_2_ within satisfactory limits but preferably keeping the blood pH between 7.3 and 7.45 and respiratory rate below 35 per minute [1, 113].

If it is not possible to ensure sufficient gas exchange within these limits, other measures may be considered, such as prone position to increase gas exchange in the lungs or extracorporeal devices, such as extracorporeal CO_2_ removal (ECCO_2_R) or extracorporeal membrane oxygenation (ECMO) [114]. Even though it is possible to preserve sufficient gas exchange within the recommended settings of PLV, some individuals may be subjected to VILI over time, especially those at risk for, or already exhibiting acute respiratory distress syndrome (ARDS) [2].

The target for MV can be either tidal volume (using constant flow) or airway pressure (using decelerating flow) but neither has been shown to be superior in terms of outcome [115]. In current computerized ventilators, a mixture of both is often used, i.e. a tidal volume is set but the ventilator uses decelerating flow to provide the required volume with as low airway pressure as possible.

In order to induce an appropriate level of VILI in mouse models, it is necessary to choose appropriate parameters for the ventilation. This includes the amount of airway pressure or tidal volume, duration of ventilation, and respiratory rate. The cumulative strain on the lung tissue is the sum of the driving pressure and respiratory rate [111].

Determining how much pressure or tidal volume to use in an experiment simulating VILI is vital to its outcome. Using too little pressure or tidal volume would result in very mild injury and would unlikely lead to significant results. On the other hand, using too much pressure or tidal volume would result in a too severe injury which is not representative of clinical reality. In either case, this would be a regrettable waste of animals. Any efforts to standardize these parameters to sufficiently recapitulate VILI in an experimental animal should therefore be of great importance, both ethically and scientifically.

Of the 99 publications on VILI we selected, 72 used tidal volume (mL/kg) as a parameter for the mechanical ventilator and 13 used pressure (cm H_2_O). Fourteen of the publications used both. Tidal volume-based terminology used in the publications varied greatly, emphasizing the need for standardized definitions. “Low” was used in 24 instances and ranged between 6 and 7.5 mL/kg, “protective” was used in 4 instances for 6–10 mL/kg and “gentle” in one at 8 mL/kg. Four publications used “normal” designation with a range of 6–10 mL/kg and “moderate” was used in 2 publications at a range of 10–12 mL/kg. In the higher ranges, “injurious” was used 5 times in the 15–25 mL/kg range and “aggressive” used in one at 15 mL/kg. “High” was used in 25 instances at a range of 12–47 mL/kg, with the most common value used being 30 mL/kg. As for the publications that used pressure target for ventilations, “low” was used at pressures of 8–15 cm H_2_O in 6 instances and “high” at 20–50 cm H_2_O in 6 instances, while “injurious” was used once at 27 cm H_2_O. The majority of articles did not designate a specific term for the tidal volume or pressure as anything other than the values listed.

PEEP is a widely used method in the clinic with the purpose of enhancing gas exchange, increasing functional residual capacity (FRC) and avoiding atelectrauma [116]. Twenty-two of the publications used 0 PEEP for their experiments, while 49 used PEEP at values from 1 to 8 cm H_2_O, with 2–3 cm H_2_O being most commonly used. Twenty-seven publications did not specify whether they used PEEP or not.

Another aspect of ventilation that needs to be considered is the breathing frequency [1]. If the same breathing frequency is maintained during normal ventilation and injurious ventilation with high tidal volume, the animal will become hyperventilated, potentially affecting circulation and acid–base homeostasis during injurious ventilation. It is therefore necessary to decrease the respiratory rate when high tidal volumes are used to avoid excessive ventilation, if the aim is mainly to study the mechanical strain.

Generally across the publications, breaths per minute were usually set lower for the higher tidal volume/pressure settings, presumably to lower the risk of excessive hyperventilation and subsequent hypocapnia and alkalosis. Considering, for this review, that tidal volume under 20 mL/kg and airway pressure 25 cm H_2_O or lower falls into the category of lower ventilation and everything above these values is considered higher, then breaths per minute in the lower category were reported in 58 instances at 40–225, with the average being at around 120. In the higher category there were reported 63 instances at 30–160 breaths per minute with an average of around 70. Nineteen of the publications did not mention breaths per minute in the ventilated animals.

During ventilator treatment in the ICU, respiratory rate is usually set between 14 and 22 breathes/min creating a ‘per minute ventilation’, with the set tidal volume, of 6–10 L/min with the aim of keeping PaCO_2_ and pH within acceptable limits. The ratio of oxygen is kept as low as possible to keep normal blood saturation within 92–98% [111].

Another parameter in creating an effective VILI model is the duration of ventilation, which should be sufficient to achieve the desired response. Ventilating the mice for too long at high pressure or tidal volume will likely result in the death of the animal [90, 117]. In the publications reviewed here, there were many instances of more than one ventilation time indicated, but for the sake of this review only the higher values were selected. In that fashion, the average time of ventilation was approximately 4.4 h. The most common ventilation time was 4 h, which was applied in 41 of the publications. Two publications reported ventilation until death of the animal, while 9 publications did not specify the duration of ventilation.

Interestingly, in all the publications that mentioned method of intubation (73 out of the 99), the mice were tracheotomized and intubated through the trachea. The reasons for tracheotomy over oral intubation of mice is presumably due to technical issues; mice are small animals and oral access to the trachea is problematic, especially in smaller mice.

### Anesthesia

VILI research is most often end-point related, meaning that the mice will be sacrificed after the ventilation. As such, general anesthesia is used and choosing the right drugs for anesthesia is crucial. Minimizing the distress and suffering of the animals is of utmost importance, both for ethical reasons, as well as for the potential influence on the experiments. A comprehensive review of mouse anesthesia was published by Gargiolu et al. [118].

In our review of the 99 publications, the most commonly used anesthesia agent was a combination of ketamine/xylazine, (40 of the publications). Ketamine concentrations, when stated, ranged from 50 to 200 mg/kg with the average being 100.1 mg/kg. Xylazine concentration ranged from 0.8 to 40 with the average being 11.8 mg/kg. Ketamine was also used in combination with acepromazine (average 1.1 mg/kg), dexmedetomidine (average 0.1 mg/kg), atropine (0.5 mg/kg and 10 µg/kg) and fentanyl (50, 90 and 120 µg/kg). Pentobarbital sodium was used in 18 of the publications at concentrations of 20–90 mg/kg (except for in one publication where it is stated as 90 µg/kg) with the average being 59.7 mg/kg. Zoletil was used in 4 publications at 5 (supplemented with 5 mg/kg of xylazine), 5, 50 (supplemented with 5 mg/kg of xylazine) and 80 mg/kg concentrations. Fentanyl/medetomidine/midazolam was used in 2 publications at 0.05/0.5/5 mg/kg concentrations, respectively, in one and at 0.075/0.75/1.5 mg/kg in the other. Celecoxib was used once at 20 or 40 mg/kg and avertin in one at 250 mg/kg. Of the 99 publications reviewed here, 18 did not specify type of anesthesia employed.

### Assays to measure VILI

**Fig. 5:**
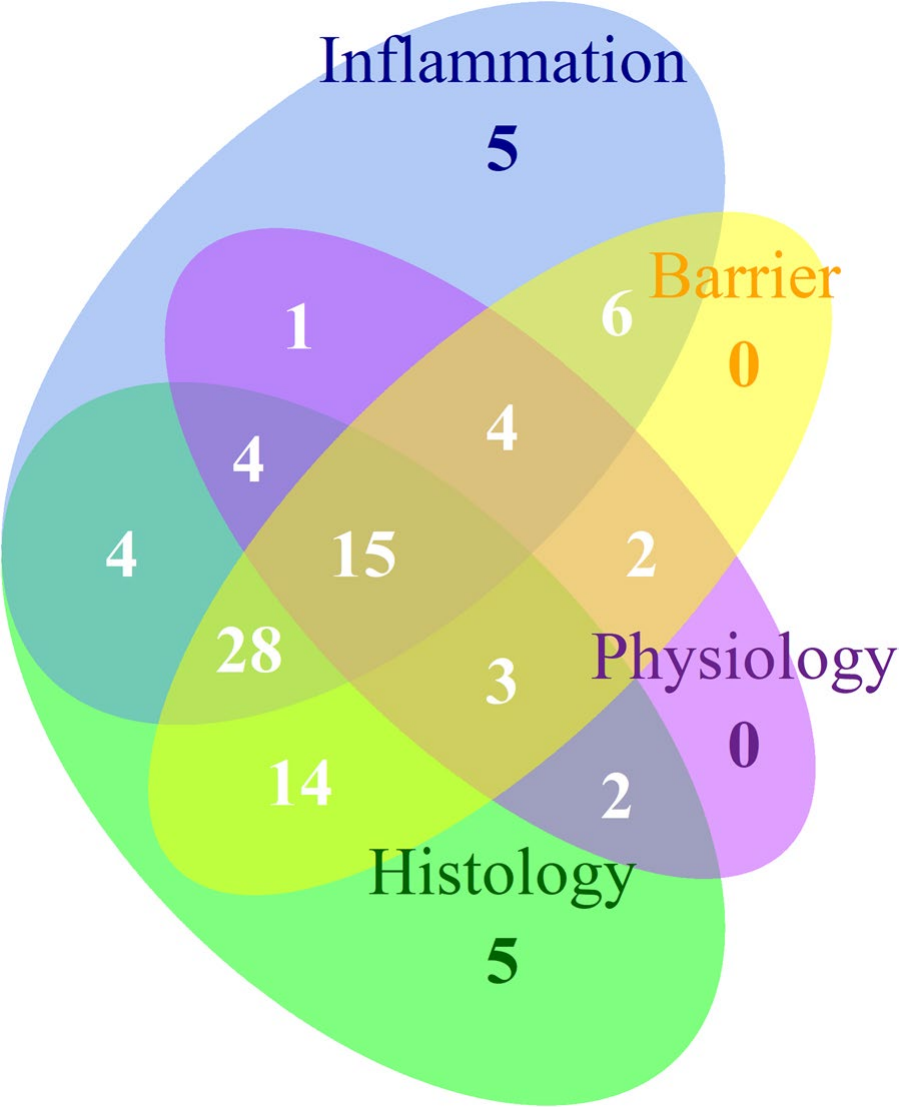
Venn diagram showing the four criteria for measuring acute lung injury in experimental animals, according to the official American Thoracic Society Workshop [7] in the reviewed publications. These criteria are measurement of inflammation, histological evidence of tissue injury, alteration of the alveolar capillary barrier and physiological dysfunction. Most often researchers measured histology, inflammation and pulmonary barrier results [24]. All four parameters were present in 15 of the 99 publications

Many of the assays used in the publications reviewed were highly specific to the research question addressed. However, there was a level of consensus in the methodologies used, including analysis of bronchial alveolar lavage fluid (BALF) (cytokine measurement and neutrophil counting), total protein concentration along with individual protein assays, cytokine increases (Most specifically IL-1β, IL-6, IL-8 and TNFα), wet-dry ratio of the lungs, H&E histology analysis, immunohistochemical measurements, and injury scoring. According to the official workshop report of the American Thoracic Society [7], the main features of experimental ALI in animals are histological evidence of tissue injury, alteration of the alveolar capillary barrier, inflammatory response, and evidence of physiological dysfunction. The reviewed VILI publications were scanned with these criteria in mind. Figure 5 illustrates how these criteria are represented in the reviewed publications and how they overlap.

Measuring histological evidence of tissue injury included counting infiltrating neutrophils and measuring lung damage in a standardized manner. Histological assessments were reported in 75 of the 99 publications.

Measurements of the alveolar capillary barrier includes measuring total protein concentration in BALF, increase in lung wet/dry ratio and Evans Blue permeability assays. Seventy-two of 99 publications employed some of these methods to measure barrier integrity.

Increased inflammatory response was reported as an increase in neutrophil or myeloperoxidase concentrations in BALF, along with increases in pro-inflammatory cytokines in lung tissue or BALF. These cytokines were often TNFα, IL-6 and IL-1β, although other cytokines were also detected. Approximately two-thirds of the publications reported their findings on inflammatory responses.

Measuring physiological dysfunction was usually in the form of PaO_2_/FIO_2_. But other factors such as lung elasticity were also measured. Thirty-one of 99 publications measured some form of physiological change due to mechanical ventilation.

Symptoms of VILI are hard to distinguish from signs of ARDS. This makes a definition of VILI difficult and it is generically classified alongside ARDS which is defined by acute onset, bilateral opacities that are consistent with pulmonary edema on chest radiographs or chest CT, respiratory failure and decreased PaO_2_/FiO_2_ ratios. Translating these definitions into mouse models is challenging but using the consensus on the main features of ALI in mice from the official American Thoracic Society Workshop [7] could be of benefit. Fifteen of the publications showed evidence of lung injury using all of the features (Fig. 5). A further 28 publications presented lung injury using evidence of an inflammatory response, barrier alteration and histology, but no physiology. This is logical given the need for quite expensive and sophisticated equipment to measure physiological parameters in the mice. Histological evidence was evaluated by paraffin embedding of lung tissue and staining. Lung injury scoring was performed in most instances. Various methods were used to measure alteration of the alveolar capillary barrier. These methods include Evans Blue permeability assays, measuring the wet/dry ratio of the lung, and measuring total protein concentration in the BALF. Inflammatory response were measured using cytokine and myeloperoxidase detection assays, most notably, ELISAs and real-time PCRs. Neutrophil infiltration in BALF and lung tissue was also measured. Physiological measurements were usually expressed in terms of PaO_2_/FiO_2_ ratio and heart rate metering.

## Conclusions

This review has revealed a large diversity in parameters used to induce VILI in mouse models over the last 10 years, especially in relation to settings and duration of ventilation required to cause sufficient injury. The variable around the experimental design and the severity and extent of the injury induced creates difficulties in interpretation and comparison of results.

Most often, researchers used the C57BL/6 inbred mouse strain (> 70%) and the majority preferred male mice. This observation was surprising as the difference between male and female C57BL/6 is most profound in their pre-experimental behaviors. Male C57BL/6 tend to be aggressive and it is more difficult to house them in a single cage. Some male mice are so aggressive that they must be kept alone in cages. However, males tend to be larger and are likely easier to handle when performing tracheotomies. Female C57BL/6 mice, on the other hand, are more docile and can easily be housed in groups of 5–10 in a single cage. Many animal facilities must take this into consideration as space may not be abundant. Another reason for the bias towards males could be the observation that males have been reported to be more susceptible to VILI and ARDS in the ICU [119]. However, a recent study performed in mice demonstrated that there is only a small difference between genders in response to mechanical ventilation, emphasizing the need to unbias the experimental setup and use both genders, in addition to easing any housing issues raised by male-only studies [120]. Other mouse strains were used in VILI studies, but to a much less extent. The second most popular mouse strain was BALB/c, then the CD-1 and ICR strains. Overall, the choice of mouse strain seems to depend mostly upon availability and ease of breeding, and is less hypothesis driven, which should be considered as the genetic background of mouse strains is highly variable and can affect the statistical outcomes of experiments.

Surprisingly, all ventilator settings differed considerably across the publications. Ventilator output was reported as tidal volume in most of the publications—72 used the term ‘tidal volume’ and 13 used the term ‘pressure’. Fourteen publications reported both tidal volume and pressure. The publications that used tidal volume as their ventilator output nominated tidal volumes of 12–47 mL/kg as “injurious”, “aggressive” or “high”, while “low”, “protective”, “moderate” and “normal” were used for pressures ranging from 6 to 12 mL/kg. In the publications that used pressure as a ventilation output, a designation of “low” was used for pressure levels of 8–15 cm H_2_O and “high” for 20–50 cm H_2_O. The margin at the higher end was considerable.

Respiratory rates differed substantially as well but tended to be lower with higher volumes/pressure. The mechanical strain the lung suffers will be the effect of tidal volume and respiratory rate, which will also affect PaCO_2_ and circulation due to differences in gas exchange and intrathoracic pressure. It is, however, difficult to define how to keep this within limits that are clinically relevant without frequent blood gas measurements, which are difficult in such small animals. However, such differences further emphasize that with standardized ventilator settings, experimental results will be more comparable.

Whether to set ventilation according to tidal volume/kg or airway pressure may be debated. Clinically, use of pressure-regulated ventilator modes has become more frequent as a part of PLV to avoid high airway pressures. In a tracheotomized mouse, the tracheal tube can easily dislodge, and the distal end becomes partially occluded by the tracheal wall producing false high airway pressures, causing the ventilator to deliver less tidal volume. In such circumstances, volume-controlled ventilation might be considered safer to ensure that the set amount of tidal volume is truly administered. This also highlights the importance of monitoring pressure and volume curves while ventilating the animal to ensure appropriate delivery of pressure, volume settings and normal airflow.

The selection of induction and maintenance anesthesia, was also quite varied among the publications. The choice of anesthesia can affect vital signs, and cardiac and respiratory function and thus, is an important aspect of the model [118, 121]. Most anesthesia selections are acceptable for mice, except for Zoletil, as it severely effects cardiorespiratory depression. The range in doses used could reflect the species, metabolism and gender of the mouse in the model. However, doses should be carefully considered to avoid causing unwanted side-effects such as altered blood pressure or obstruction. Ketamine/xylazine, the barbiturate pentobarbital, and combination anesthesia of ketamine/medetomidine and benzodiazepines/opioids, as used in the studies here, are reported to be effective in mouse respiratory models. Given that body temperature, respiratory and heart rates, and PaO_2_ are frequently monitored and shown to be relatively stable, comparison between studies should be achievable.

Interestingly, most of the articles do not provide information about monitoring the vital signs of the mouse and thus lack a criterion for assessing if the mouse is alive or not during ventilation, as this can be difficult to assess with the naked eye. Ventilating a deceased mouse could affect the results drastically and so a set of criteria, including, for example, heart rate monitoring with an ECG, to judge at which time point the mouse is considered deceased, is preferable.

This review provides an overview of experiments conducted over the last 10 years on VILI mouse models, especially in relation to settings and duration of ventilation considered appropriate to cause sufficient injury. Our results show a wide variety in these models, causing uncertainty in the amount and degree of VILI produced in each model and therefore difficulties in interpreting results and comparing them between studies. We do not offer a solution to this problem but the information highlighting this here could possibly raise awareness of this situation and encourage a more standardized approach to certain research questions. A consensus needs to be reached regarding ventilator settings, to create a more standardized model in order to facilitate effective transfer of knowledge from in vivo models to the clinical settings.

The confounding factors leading to VILI are difficult to replicate in the mouse model, and here we have highlighted the diversity of parameters that need to be set. Many of the publications we reviewed employed similar methods for inducing VILI, but the intensity of the ventilator settings differed considerably, which makes it difficult to predict which settings are appropriate.

In summary, stringent guidelines and a standardized model is needed to be able to effectively transfer the knowledge from the in vivo model to the clinical setting.

## Supplementary Information

**Additional file 1.** Supplemental table 1.

## Data Availability

All articles reviewed here were found using a PubMed search with the search title “ventilator induced lung injury mouse model”, filtering the publication year from 2010 to 2020. From the 372 matching hits, we concentrated on models where mice were mechanically ventilated, leaving a pool of 99 publications.

## Abbreviations

ARDS: Acute respiratory distress syndrome
BALF: Bronchoalveolar lavage fluid
CPAD: Cyclical pressure air–liquid interface device
CT: Computed tomography
ECCO_2_R: Extracorporeal CO_2_ removal
ECG: Electrocardiogram
ECMO: Extracorporeal membrane oxygenation
FRC: Functional residual capacity
FIO_2_: Fraction of inspired oxygen
H&E: Hemotoxylin and eosin
ICU: Intensive care unit
IL: Interleukin
MV: Mechanical ventilation
PaCO_2_: Partial pressure of carbon dioxide
PaO_2_: Partial pressure of oxygen
PEEP: Positive end-expiratory pressure
PLV: Protective lung ventilation
TNF: Tumor necrosis factor
VILI: Ventilator-induced lung injury
